# Clinical guideline for the diagnosis and treatment of cutaneous warts (2022)

**DOI:** 10.1111/jebm.12494

**Published:** 2022-09-18

**Authors:** Peiyao Zhu, Rui‐Qun Qi, Yang Yang, Wei Huo, Yuqing Zhang, Li He, Gang Wang, Jinhua Xu, Furen Zhang, Rongya Yang, Ping Tu, Lin Ma, Quanzhong Liu, Yuzhen Li, Heng Gu, Bo Cheng, Xiang Chen, Aijun Chen, Shengxiang Xiao, Hongzhong Jin, Junling Zhang, Shanshan Li, Zhirong Yao, Weihua Pan, Huilan Yang, Zhu Shen, Hao Cheng, Ping Song, Lingyu Fu, Hongxiang Chen, Songmei Geng, Kang Zeng, Jianjian Wang, Juan Tao, Yaolong Chen, Xiuli Wang, Xing‐Hua Gao

**Affiliations:** ^1^ Department of Dermatology The First Hospital of China Medical University Heping District Shenyang P.R. China; ^2^ NHC Key Laboratory of Immunodermatology, China Medical University Heping District Shenyang P.R. China; ^3^ Key Laboratory of Immunodermatology, China Medical University Ministry of Education Heping District Shenyang P.R. China; ^4^ National and Local Joint Engineering Research Center of Immunodermatological Theranostics Heping District Shenyang P.R. China; ^5^ Department of Clinical Epidemiology and Evidence‐Based Medicine The First Hospital of China Medical University Heping District Shenyang P.R. China; ^6^ Department of Dermatology First Affiliated Hospital of Kunming Medical University Kunming P.R. China; ^7^ Department of Dermatology Xijing Hospital Fourth Military Medical University Xi'an, Shaanxi P. R. China; ^8^ Department of Dermatology Huashan Hospital Fudan University Shanghai P.R. China; ^9^ Shandong Provincial Hospital for Skin Diseases & Shandong Provincial Institute of Dermatology and Venereology Shandong First Medical University & Shandong Academy of Medical Sciences Jinan P.R. China; ^10^ Department of Dermatology General Hospital of Beijing Military Command of PLA Dongcheng District Beijing P.R. China; ^11^ Department of Dermatology and Venerology Peking University First Hospital Beijing P.R. China; ^12^ Department of Dermatology Beijing Children's Hospital Capital Medical University National Center for Children's Health Beijing P.R. China; ^13^ Department of Dermatology Tianjin Medical University General Hospital Tianjin P.R. China; ^14^ Department of Dermatology Second Affiliated Hospital of Harbin Medical University Harbin P.R. China; ^15^ Institute of Dermatology Chinese Academy of Medical Sciences and Peking Union Medical College Nanjing P.R. China; ^16^ Department of Dermatology The First Affiliated Hospital of Fujian Medical University Fuzhou P.R. China; ^17^ Department of Dermatology Xiangya Hospital Central South University Changsha P.R. China; ^18^ Department of Dermatology The First Affiliated Hospital of Chongqing Medical University Chongqing P.R. China; ^19^ Department of Dermatology The Second Affiliated Hospital School of Medicine Xi'an Jiaotong University Xi'an P.R. China; ^20^ Department of Dermatology Peking Union Medical College Hospital Chinese Academy of Medical Sciences and Peking Union Medical College Dongcheng District Beijing P.R. China; ^21^ Department of Dermatology Tianjin Academy of Traditional Chinese Medicine Affiliated Hospital Tianjin P.R. China; ^22^ Department of Dermatology The First Hospital of Jilin University Changchun Jilin Province P.R. China; ^23^ Department of Dermatology Xinhua Hospital Shanghai Jiao Tong University School of Medicine Shanghai P.R. China; ^24^ Department of Dermatology Shanghai Key Laboratory of Molecular Medical Mycology Second Affiliated Hospital of Naval Medical University Shanghai P.R. China; ^25^ Department of Dermatology General Hospital of Southern Theatre Command of PLA Guangzhou P.R. China; ^26^ Department of Dermatology Institute of Dermatology and Venereology Sichuan Academy of Medical Sciences and Sichuan Provincial People's Hospital Chengdu P.R. China; ^27^ Department of Dermatology and Venereology Sir Run Run Shaw Hospital School of Medicine Zhejiang University Hangzhou P.R. China; ^28^ Department of Dermatology Guang'anmen Hospital China Academy of Chinese Medical Sciences Beijing P.R. China; ^29^ Department of Clinical Epidemiology and Evidence‐Based Medicine The First Hospital of China Medical University Heping District Shenyang P.R. China; ^30^ Department of Dermatology Union Hospital Tongji Medical College Huazhong University of Science and Technology Wuhan P.R. China; ^31^ Department of Dermatology The Second Affiliated Hospital of Xi'an Jiaotong University Xi'an Shaanxi P.R. China; ^32^ Department of Dermatology Nanfang Hospital Southern Medical University Guangzhou P.R. China; ^33^ Evidence‐Based Medicine Center School of Basic Medical Sciences Lanzhou University Lanzhou P.R. China; ^34^ World Health Organization Collaborating Center for Guideline Implementation and Knowledge Translation Lanzhou P.R. China; ^35^ GIN Asia Lanzhou P.R. China; ^36^ Institute of Photomedicine Shanghai Skin Disease Hospital School of Medicine Tongji University Shanghai P.R. China

**Keywords:** common wart, condyloma acuminatum, epidermodysplasia verruciformis, flat wart, plantar wart

## Abstract

**Aim:**

Cutaneous warts caused by human papillomavirus are benign proliferative lesions that occur at any ages in human lives. Updated, comprehensive and systematic evidence‐based guidelines to guide clinical practice are urgently needed.

**Methods:**

We collaborated with multidisciplinary experts to formulate this guideline based on evidences of already published literature, focusing on 13 clinical questions elected by a panel of experts. We adopted Grading of Recommendations Assessment, Development and Evaluation (GRADE) system to form classification of recommendations as well as the improved Delphi method to retain respective recommendations with a consensus degree of over 80%.

**Results:**

Our guideline covered aspects of the diagnosis and treatment of cutaneous warts such as diagnostic gold standard, transmission routes, laboratory tests, treatment principle, clinical cure criterion, definitions, and treatments of common warts, flat warts, plantar warts, condyloma acuminatum, and epidermodysplasia verruciformis. Recommendations about special population such as children and pregnant women are also listed. In total, 49 recommendations have been obtained.

**Conclusions:**

It is a comprehensive and systematic evidence‐based guideline and we hope this guideline could systematically and effectively guide the clinical practice of cutaneous warts and improve the overall levels of medical services.

## 1 INTRODUCTION

Cutaneous warts are proliferative diseases caused by human papillomavirus (HPV) infection of keratinocytes. Viral warts are common with a prevalence rate of 7–12%.^1^, ^2^ HPV is a double‐stranded DNA virus with more than 200 types being identified. HPVs can be grossly divided into high‐risk types and low‐risk types for their carcinogenic potentials. The life cycle of HPV is closely associated with the proliferation and differentiation of epithelium. Cutaneous HPV infection commonly manifests as warts including flat warts (verruca plana, on hands and face), common warts (verruca vulgaris), planta warts (verruca plantaris, on soles of feet), and condyloma acuminatum (anogenital warts, on genitalia, anus or perianal area).^3^ Most cutaneous HPV infection leads to benign proliferative lesions, while rarely develops into cutaneous cancers such as squamous cell carcinoma.^4^ Appropriate measures for prevention, diagnosis, treatment, and long‐time management of cutaneous HPV infection are mandatory for dermatologists, pediatricians, urinologists, gynecologists, and general practitioners.

Albeit there are several major guidelines or consensus for cutaneous warts,^5^, ^6^, ^7^, ^8^, ^9^, ^10^, ^11^, ^12^, ^13^ a comprehensive and systematically produced guidance for management of cutaneous HPV infection including flat warts, common warts, plantar warts, and anogenital warts is missing. We summarized the recent clinical progress and incorporated recommendations based on evidence and expert consensus, dedicated to provide a general guideline for the prevention, diagnosis, treatment, and long‐term management of cutaneous warts.

## 2 METHODS

### 2.1 Scope and registration of the guideline

The target population of this guideline are patients with cutaneous warts including common warts, flat warts, plantar warts, condyloma acuminatum (CA), and epidermodysplasia verruciformis (EV) , caused by HPV infection. The content covers aspects such as screening, diagnosis, treatment, and prevention. Special populations such as children and pregnant women are also taken into account. The guidelines are applicable to medical institutions at all levels. Target implementing agencies are medical institutions and health management departments that provide health care services to the target population. The main users of the guidelines are medical workers in the departments of Dermatology and Venereology, Obstetrics and Gynecology, and Preventive Health Care. Proctologists and infectiologists are practitioners of this guideline as well.

This guideline has been bilingually registered on the International Practice Guidelines Registry Platform (http://www.guidelines‐registry.cn) with registration number IPGRP‐2020CN078 on June 9, 2020.

### 2.2 Guideline working group

This guideline was launched and formulated by the Chinese Society of Dermatology on June 1, 2020. Methodological support was provided by the WHO Collaborating Centre for Guideline Implementation and Knowledge Translation, GRADE China Center, and Evidence‐Based Medicine Center of Lanzhou University. The guideline working group consisted of five groups: (I) a guideline steering committee, consisted of 9 senior clinical experts and methodologists; (II) a consensus expert group, consisted of 32 panelists from professional field of dermatology; (III) a secretarial group, who was fully responsible for the coordination and management of the guide, the collection and sorting of evidence and data, the arrangement and recording of various tasks, the contact and communication of relevant experts, and all issues not covered by other working groups; (IV) an evidence evaluation group consisted of 33 members, which was responsible for finding, collecting, evaluating and synthesizing relevant evidence, and applying GRADE grading system,^14^ making decision tables, and preparing for expert consensus; and (V) an external review group consisted of 10 members, which was mainly responsible for reviewing the first draft of the guideline, putting forward comments and suggestions, and its work was intended to be completed before the guideline was officially released.

### 2.3 Collection and determination of clinical questions

We used predesigned questionnaires to collect clinical questions. The responders of the survey were clinical doctors in dermatology. Meanwhile, by referring to the relevant guidelines and systematic reviews of HPV‐related skin diseases, we collected potential clinical questions. We distributed original questionnaires to 20 dermatologists in the first round and modified questionnaires to 50 dermatologists in the second round of the survey with both 100% response ratios. Through two rounds of questionnaire surveys, based on the scoring results by order of importance of the clinical issues, 13 clinical questions were finally included in this guideline.

### 2.4 Evidence collection

**FIGURE 1:**
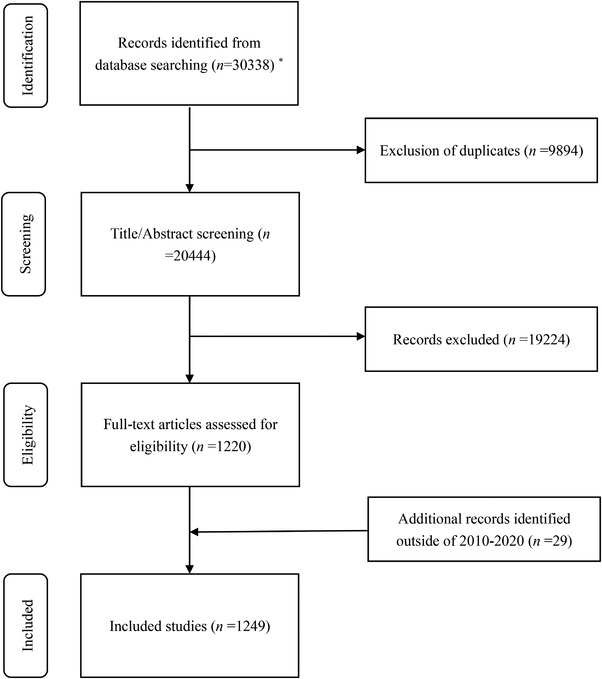
Flow chart of literature screening *Results of searching: Medline (*n* = 4687), Embase (*n* = 3312), Cochrane Library (*n* = 1313), Epistemonikos (*n* = 215), CBM (*n* = 8334), Wanfang (*n* = 8768), and CNKI (*n* = 3709)

This guideline collected evidence from systematic reviews, meta‐analysis, and network meta‐analysis. The search terms included “Warts” or “Condylomata Acuminata” or “Epidermodysplasia Verruciformis” or “verruca vulgaris” or “verruca plantaris” in both Chinese and English respectively from 2010 to 2020, in the order of title or abstract. Search databases included Medline, Embase, Cochrane Library, Epistemonikos, China Biology Medicine (CBM), Wanfang, and China National Knowledge Infrastructure (CNKI). In cases where no systematic reviews or meta‐analysis were available, we systematically searched the database, generating a systematic review according to the original research data or incorporating it into the original research to construct an evidence body. A flow chart of literature screening was shown in Figure 1. Among the 49 recommendations of the 13 clinical questions, 43 recommendations were based on existing reviews while 6 recommendations were based on newly generated reviews or evidence bodies of original research. A total of 29 new reviews were generated for this guideline. The information from the included research papers was extracted according to the predesigned data extraction table. The screening and information extraction of each document was done independently by two groups of members. A third party was consulted if there were discrepancies.

### 2.5 Evidence assessment and grading

We used the systematic review bias risk assessment tool, A MeaSurement Tool to Assess systematic Reviews (AMSTAR) scale, to evaluate the bias risk of the included systematic reviews, meta‐analysis, and network meta‐analysis.^15^ We also used the Cochrane risk of bias (ROB) assessment tool (for randomized controlled trials), diagnostic accuracy research quality assessment tool (Quality Assessment of Diagnostic Accuracy Studies, QUADAS‐2, for diagnostic tests), and Newcastle‐Ottawa Scale (NOS, for cohort studies and case‐control studies) for methodological quality evaluation of respective types of original research.^16^, ^17^, ^18^ The evaluation process was completed by two members independently and if there was a disagreement, they would discuss it together or consult a third party to resolve it. The GRADE method (Table 1) was used to evaluate the quality of the evidence, and the quality of the evidence was divided into four levels: high, moderate, low, and very low.^14^, ^19^, ^20^ They had been presented in an evidence summary table.

**TABLE 1:** Strength of recommendations and levels of evidences

Item	Definition
Strength of recommendations	
Strong (1)	It clearly shows that the intervention does more harm than good or does more good than harm.
Weak (2)	The benefits and harms are uncertain or the quality of the evidence shows comparable benefits and harms.
Levels of evidences	
High (A)	We are very confident that the observed value is close to the true value.
Moderate (B)	We have moderate confidence in the observed value: the observed value is likely to be close to the true value, but it might be substantially different.
Low (C)	We have limited confidence in the observed value: the observed value may be substantially different from the true value.
Very low (D)	We have little confidence in the observed value: the observed value can be substantially different from the true value.

### 2.6 Formulation of recommendations

**FIGURE 2:**
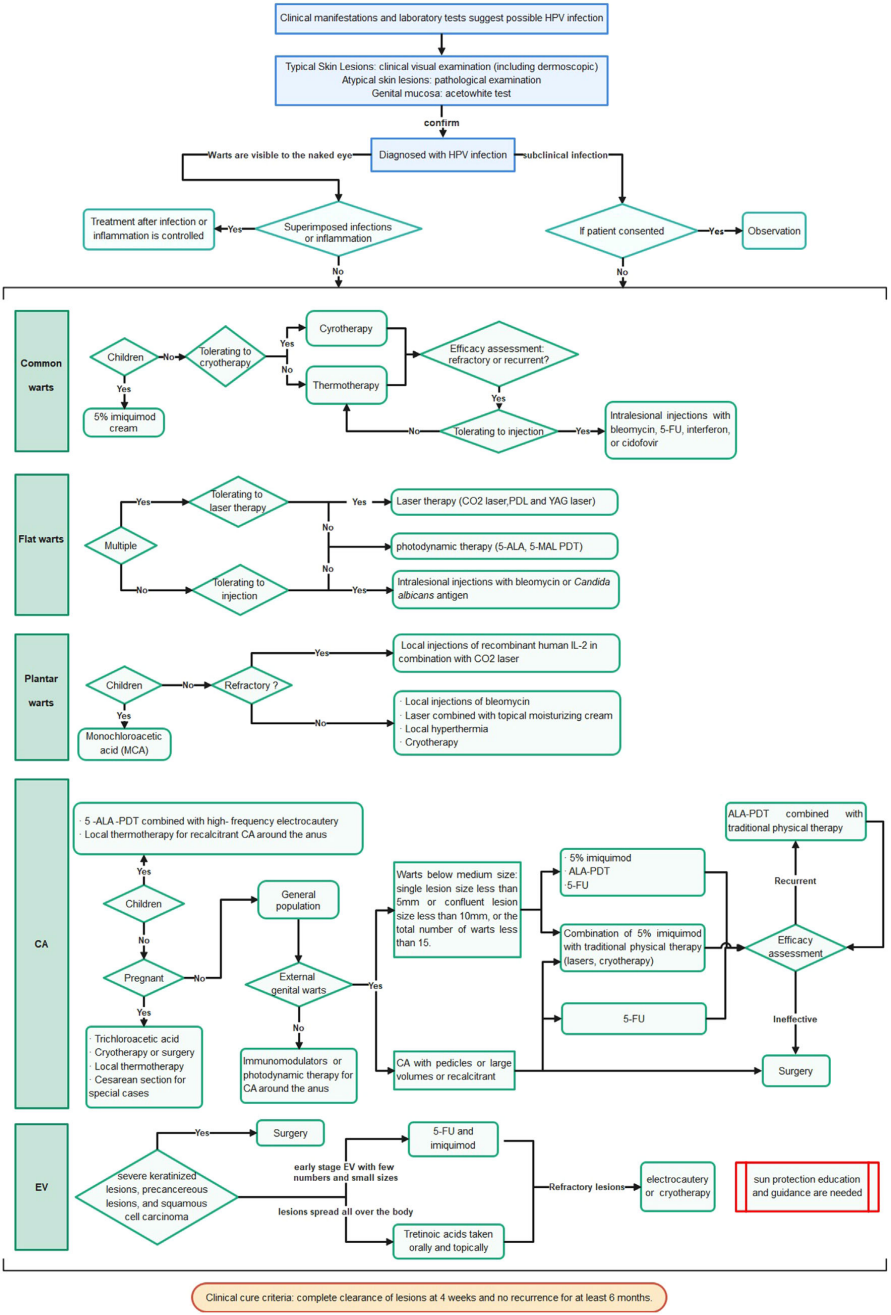
A diagram of the diagnosis and treatment of cutaneous warts

After four to five rounds of revisions, 60 relevant recommendations and the supporting materials for recommendations were initially determined. The secretary group made the GRADE decision‐making table and reached a consensus on recommendations through 2–3 rounds of surveys using the improved Delphi method.^21^ Upon evaluation by 27 expert panels and after considering the patient's preferences and values, and the costs, benefits, and harms of the interventions, 49 recommendations were finally formed with a consensus degree of over 80% and the corresponding recommendation basis was included. We referenced Reporting Items for Practice Guidelines in Healthcare (RIGHT) to write this guideline. The expert panel approved a diagram of management of patients with cutaneous warts (Figure 2). Plan for updates on recommendations of this guideline will be initiated around 2025 according to the requirement by the international guide.^22^, ^23^

## 3 RESULTS

**Question 1: What is the gold standard for the diagnosis of skin/mucosal HPV infection?**

**Recommendation**:

1. Typical viral warts can be diagnosed by clinical visual examination. Pathological examination and HPV genotyping are recommended in cases of atypical lesions (suspected precancerous lesions or cancer) and in cases where the diagnosis is uncertain. (1C)
2. A 3−5% acetowhite test is suggested in the diagnosis of HPV infection in the genital mucosa. (2C)

**Summary of the evidence**:

Viral warts are generally diagnosed by visual recognition. However, the identification of atypical skin lesions should be evaluated by pathological examination and HPV genotype testing. For early genital mucosa viral warts, the application of 3–5% acetic acid can help in the detection of subclinical skin lesions.^5^, ^6^, ^7^, ^8^, ^24^

A case series study^25^ which examined 51 suspected men with CA by pathological examination and colposcopy showed that the accuracy of histopathological examination in the diagnosis of CA was higher than that of colposcopy (95.60% vs. 88.20%). A case series described that infection by different HPV types contributed to varied histological patterns in association with clinical types.^26^ A diagnostic accuracy test^27^ showed that the HPV‐positive rate of the histopathologically diagnosed CA was 95.00%. The sensitivity and specificity for the prediction of HPV 6/11 by pathological examination were 43.60–46.60% and 64.70–71.70%, respectively. A case‐series study^28^ showed that all 116 hyperplastic warts were positive, and 57.70% of flat warts in the moist area were positive, while those in the dry area were practically negative when 5% acetic acid was applied. Two clinical studies revealed that in patients with CA, the accuracy of 5% acetic acid white test was 55.30% while in patients with subclinical HPV infection, the sensitivity and specificity of the acetowhite test were 92.30% and 58.20%, respectively.^29^, ^30^

**Question 2: What are the transmission routes of HPV infection of skin or mucosa?**

**Recommendation**:

1. Sexual and mother‐to‐fetus vertical transmissions are the main transmissions route of HPV to cause CA. (1C)
2. Virus transmission by skin contact, hand spreading, and contact with underwear or inanimate objects are responsible for common warts, plane warts, and plantar warts. (2D)
3. High‐temperature evaporation treatment, i.e., CO_2_ lasers, produces smog from the destructed HPV containing lesions that could transmit HPVs. (2B)

**Summary of the evidence**:

HPV has more than 200 types and causes multiple diseases including cutaneous and anogenital warts, cervical cancer, and anal cancer in men and women. Understanding the transmission routes of HPV can lead to better prevention for it.^31^

A cross‐sectional study^32^ found that 64.29% (169/263) couples, of whom at least one person was infected and 42.01% of partners harbored the same HPV type (95% CI [36, 47]). The high degree of concordance suggests a high probability of sexual transmission. A cohort study^33^ showed that the mother‐to‐fetus vertical transmission rate of HPV was 27.66%. There was no significant difference in infants’ HPV infection rate between vaginal delivery and cesarean delivery (25.71% vs. 28.81%).

A systematic review in 2020^34^ showed that mucosa of the upper respiratory tract (nose, mouth, pharynx) is a more common site with warts in CO_2_ laser users compared to the normal population, as well as to those who don't use LEEP (Loop Electrosurgical Excision Procedure) or CO_2_ lasers (0.60–3.40% vs. 5.10–12.90%) (OR = 5.75, 95% CI [1.55, 21.38], *p* < 0.001). Therefore, local exhaust ventilation such as smoke evacuators was recommended when performing laser or electrosurgical treatments for patients with warts.^35^

**Question 3: What are the laboratory tests for cutaneous warts?**

**Recommendation**:

1. Acetowhite test is recommended for the diagnosis of subclinical CA. (1C)
2. In cases where identification of HPV types is required, noninvasive sampling by skin swabbing is recommended for HPV testing, resection or clamping of the warty tissue may be necessary in the case.(1C)
3. Dermoscopy may aid in the diagnosis of viral warts. (2D)
4. For vulvar CA harboring high‐risk HPVs, cervical HPV test is suggested. (2B)

**Summary of the evidence**:

Albeit controversial for the recommendation of acetowhite test for the diagnosis of CA, it is still clinically used as an economical and convenient inspection method. Several studies^28^, ^29^, ^30^, ^36^ have shown the efficacy of acetowhite test to detect CA, including those with inconspicuous clinical appearance.

A case‐series study^37^ reported that PCR detection from samples of the resected wart and the wart swab yielded HPV‐positive rates of 92.00% and 88.00%, respectively (*p >* 0.05). Another case‐series study^38^ showed that 25 swabbed samples possess identical HPV types to the biopsy counterpart with 96.00% sensitivity, a result validating that wart swabs can be reliably used for HPV typing sampling. A self‐controlled experiment^39^ showed that both two sampling methods, swabbing the surface of the lesion and taking a shave biopsy, reached a high agreement for detection of HPV DNA in CA (87.80% agreement) and penile intraepithelial neoplasia (100% agreement). However, the agreement in these two methods was low to moderate for detecting most individual HPV types, thus a combination of biopsy with swabbing may provide additional information for HPV genotyping.

A case‐series study^40^ including 132 patients with a total of 220 suspected CA lesions showed that the positive rate of dermatoscopic diagnosis was higher than that of a physician's visual diagnosis. A study^41^ reported that dermoscopy provided a higher positive rate and superiority in observing tiny warts than visual observation (*p* < 0.01). This method possessed the advantages of high sensitivity, quick and accurate diagnosis, and noninvasiveness. However, it has limitations for its inaccessibility in deep urethral orifice and skin wrinkles.

Two cohort studies^42^, ^43^ showed a higher risk of cervical cancer, CIN (Cervical Intraepithelial Neoplasia), and cervical cancer in situ in female patients with CA. In 2012, a study^44^ reported that the rate of cervical high‐risk HPV infection was significantly higher in women with CA than in the general population.

**Question 4: What is the treatment principle for cutaneous warts?**

**Recommendation**:

1. Remove the wart as early as possible and eliminate the subclinical infection around the wart as much as possible to reduce or prevent recurrence. (1C)
2. Superimposed infections and inflammation should be controlled before treating CA lesions. (1C)
3. Decision on the treatment of genital warts in pregnant women should be based on the size of warts and the impact on the fetus. (2D)
4. Precaution to avoid contact with flowing particles should be taken during the evaporating surgical treatment of warts. (2C)

**Summary of the evidence**:

The primary goal of treatment is to remove warts and improve the presenting symptoms. According to the published guidelines,^5^, ^13^, ^45^ most patients' warts disappear after treatment while the recurrences are frequent. Genital warts may heal, remain unchanged, or increase in number and size in untreated patients. Treatment may weaken the infectivity of HPV, but may not necessarily eradicate HPV. A guideline of the Chinese Society of Dermatology in 2021^9^ suggested that visible genital warts should be treated. For subclinical infections, laser, cryotherapy, topical imiquimod, photodynamic therapy (extending to 1 cm around warts) should be appropriately implemented to reduce the rate of recurrence.

So far, there is no effective anti‐HPV drug to clear HPV infection. Surgery and physical therapy can remove visible warts. An expert consensus in 2017 in China^46^ indicated that cytological examination should be performed before the treatment of vaginal and cervical CA. And if necessary, colposcopic biopsy should be performed to exclude the precancerous and cancerous lesions of the vagina and cervix.

A guideline from the Chinese Society of Dermatology in 2015^47^ stated that patients with CA may be complicated with other sexually transmitted diseases. In such cases, the inflammation or other superimposed infections should be controlled first, to avoid spreading skin lesions after treatment.

The numbers and sizes of genital warts may increase with the progress of the pregnancy. The safety profile of topical podophyllotoxin and imiquimod during pregnancy has not been established and thus prohibited. Cryotherapy, surgery, and trichloracetic acid for genital warts are applicable to pregnant women. Pregnant women should be treated appropriately to ensure the safety of the fetus. China's 2014 Guidelines for Diagnosis and Treatment of CA^10^ and an expert consensus in 2017^11^ both recommended that pregnant women infected with CA should be treated as early as possible. Genital warts rarely affect delivery, and their spontaneous resolution was common during the puerperium. In 2019, a guideline from the Infection and Sexual Health Clinical Research Center of the Institute of Global Health, University of London, UK^6^ stated that for small, slow‐growing warts which did not affect pregnancy and delivery, the treatment of genital warts could be postponed until after childbirth. If there are warts that might block the birth canal, pelvic outlet obstruction, or vaginal delivery, a joint consultation with specialists in obstetrics and gynecology, neonatology, and venerology is necessary.^9^ The spouse or partner who had sexual contact with the patient should be examined and they need to be treated if with lesions. Avoidance of intercourse is recommended during treatment. Choices of treatment methods should be based on considerations of the size, location, age, and other factors of the skin lesions. Toxic drugs or methods prone to scarring were not recommended.

During the surgical treatment of HPV‐related lesions, especially when using smoke‐producing surgical treatment methods (laser or electrosurgery), it was recommended to comply with laser safety regulations and hygiene guidelines to protect patients and physicians from contacting infectious particles.^5^

**Question 5: What is the clinical criterion for the cure of warts?**

**Recommendation**:

1. The clinical criteria for cure of warts are complete clearance of lesions at 4 weeks and no recurrence for at least 6 months. (1B)

**Summary of the evidence**:

According to the current literature, there has been no uniform definition of clinical criteria for the cure of HPV infection. A systematic review published in 2017^48^ referred that short‐term complete clearance referred to complete clearance of lesions at 4 weeks (± 4 weeks) at the end of treatment (EOT), intermediate‐term complete clearance referred to complete clearance of lesions at 16 weeks (± 8 weeks) at the EOT, and long‐term clearance referred to complete clearance of lesions at 12 months (± 6 months) at the EOT. Intermediate‐term recurrence referred to recurrence of lesions at 16 weeks (± 8 weeks) at EOT in patients who had a complete clearance at 4 weeks (± 4 weeks) at EOT. Long‐term recurrence referred to recurrence of lesions at 12 months (± 6 months) at EOT in patients who had a complete clearance at 4 weeks (± 4 weeks) at EOT. Another systematic review in 2017^49^ reported that, in HIV‐positive patients with genital warts, short‐term complete clearance referred to complete clearance of lesions at 4 weeks (after EOT), intermediate‐term complete clearance referred to complete clearance of lesions at 24 weeks (± 16 weeks) at EOT, and long‐term clearance referred to complete clearance of lesions at 12 months (± 2 months) at EOT. Intermediate‐term and long‐term recurrence respectively referred to recurrence of lesions at 24 weeks (± 16 weeks) and 12 months (± 2 months) after EOT in patients who had a complete clearance at EOT. For other warts, an RCT^50^ in 2020 including recalcitrant common warts and a study protocol for a single‐center randomized controlled trial^51^ in 2020 including common warts, plantar warts, flat warts, and filiform warts both stated that the recurrence rate was assessed at 6 months after enrolment.

**Question 6: How to define multiple, recurrent, and refractory cutaneous warts?**

**Recommendation**:

1. Multiple warts are defined as a patient with two or more than two warts. (2D)
2. Recurrent warts are defined as warts that appear near the original site of warts, which have been completely cleared. The intermediate‐term recurrence of warts is 4 months (± 2 months) by the end of treatment, and the long‐term recurrence is 12 months (± 6 months) by the end of treatment. (2B)
3. Refractory warts are defined as warts that last for at least 2 years with poor response to more than two traditional treatment options. (2B)

**Summary of the evidence**:

There has been no uniform definition of multiple/generalized, recurrent, and refractory warts thus far. Clinicians mostly classify them according to their understanding.

In a cohort study,^52^ albeit the authors didn't explicitly define multiple warts, the presumptive were those patients with more than one warty lesion. In a 2016 case report,^53^ the generalized wart was defined as diffuse cutaneous warts over 20 in number that is distributed in more than one area of the body.

In 1989, a nonrandomized controlled study^54^ indicated that recurrent warts were those that appeared near the original sites with warts that had been cleared completely. Two systematic reviews in 2017^48^, ^49^ showed that intermediate‐term recurrence of genital warts was 4 months (± 2 months) after the EOT, and long‐term recurrence was 12 months (± 6 months) after the EOT (the skin lesions were completely cleared at the EOT). Two Chinese expert consensuses in 2015 and 2017^11^, ^12^ stated that the recurrence of CA mostly occurred in 3–6 months after treatment, and most often occurred in the first 3 months. If there were no recurrence 6 months after treatment, the chance of recurrence was low.

In a nonrandomized controlled study,^55^ refractory warts were defined as those who failed to respond to two traditional treatments or warts that lasted for over 2 years. A cohort study^56^ showed warts were more persistent and refractory to treatment in organ transplant recipients. In an RCT in 2018,^57^ the included refractory warts were those that lasted for more than 2 years and failed response to more than two treatment methods (laser surgery, electrosurgery, curettage, liquid nitrogen freezing therapy, and topical salicylic acid treatment). In an observational study,^58^ refractory warts were those resistant to conventional treatments and there was no improvement after trying different methods.

**Question 7: What are the recommended clinical treatment methods for common warts?**

**Recommendation**:

1. Local injections with bleomycin, 5‐Fluorouracil (5‐FU), and cidofovir are suggested for refractory and recurrent common warts. Local adverse reactions of intralesional injection therapy include pain, burning, itching, erythema during the procedures, and postinflammatory pigmentation. (2B)
2. Cryotherapy is recommended for common warts. However, patients receiving cryotherapy need to tolerate treatment‐related pain and, may experience other side effects, such as posttreatment scarring and hyper/hypopigmentation. (1B)
3. Thermotherapy is suggested for patients with common warts, especially multiple warts, who cannot tolerate local injection and cryotherapy. The recommended treatment temperature is on average at 44°C over the lesion, and the treatment time lasts for 30 min. Repeated treatments are required. Common adverse reactions include burning sensation, occasional heat‐induced blisters, and postinflammatory pigmentation. (2C)

**Summary of the evidence**:

Common warts are mostly caused by HPV type 2. Choices for their treatment should be based on specific conditions.^59^

A case‐series study^60^ showed that after an average of 2.61 treatment cycles with intralesional injection of bleomycin (therapeutic dose at 3 U/ml and treatment interval at 3–4 weeks), all of the 250 periungual and subungual warts in 80 patients of whom 26 (32.50%) patients were either with no response to or recurrence after previous treatments were cleared and 65 (81.25%) patients experienced moderate pain during the treatment sessions, 155 (62.00%) treatment sites had transit dyspigmentation, and 3 (1.20%) treatment sites experienced reversible necrosis. An RCT^61^ including 42 patients with multiple warts (common warts and plantar warts) compared the bleomycin microneedle patch treatment with cryotherapy and their effective rates were respectively 55.71% and 55.85%, but patients were more tolerable to microneedle patch for lesser pain during the treatment.

A prospective study^62^ reported that the cure rate of common warts with the injection of 5‐FU, lidocaine, and epinephrine mixture (50 mg/ml 5‐FU plus with lidocaine and epinephrine mixture at a ratio of 4:1) was higher than that of saline control (64.70% vs. 35.30%, *p*<0.05). There was no significant difference in the incidence of systemic adverse reactions and treatment‐related side effects between the two groups. A case‐series study^63^ included 280 patients with multiple and recalcitrant cutaneous warts (common and mosaic warts) and without any success, they had received at least two other treatments for their lesions. The result showed that lesional injection with 15 mg/ml cidofovir once a month, on average of 3.2 sessions, cleared relapsed and refractory warts in 276 of 280 patients.

An observational study on 90 patients^64^ showed that the overall success rate was 64.44% in treating warts on hands and feet with cryotherapy. The effectiveness of liquid nitrogen cryotherapy for common warts depended on factors including the duration of warts, the number of warts, and the repeated times of treatments. Warts treated by cryotherapy once a week had faster recovery than once every 2–3 weeks while the overall cure rate depended on the total repeated times of treatments rather than the time interval. A meta‐analysis on one nonrandomized controlled trial^65^ and two RCTs^61^, ^66^ showed that the effectiveness of liquid nitrogen cryotherapy in the treatment of common warts was significantly higher than noncryotherapy methods (*I* ^2^ = 90%, RR = 2.01, 95% CI [1.02, 3.97], *p* = 0.04), as topical trichloroacetic acid and intralesional injection of *Candida* antigen. A cohort study^67^ concluded that shorter freezing time (10 s) and interval (2 weeks) of cryotherapy was more effective than longer freezing time (20 s) and intervals (4 weeks), for treating common viral warts on hand and foot.

A case‐series study^68^ performed local hyperthermia (once a day for 30 min, for 5 consecutive days, temperature applied was determined by tolerance of the patients, on average, temperatures applied to hand warts was 43.5°C, while that for foot warts was 45.3°C) on patients with common warts who had not received local or systemic treatment in the past 3 months. For patients with multiple lesions, only one target lesion was selected for local hyperthermia. After the treatment, the patients were followed up monthly. After 3 months of follow‐up, the total cure rate was 53.85%, and the cure rate for the foot (65.22%) was higher than the hand cure rate (37.50%). Treatment response was not affected by number, gender, and age. All patients had tolerable burning sensations during treatment.

**Question 8: What are the recommended clinical treatment methods for flat warts, including generalized, recurrent, and refractory conditions?**

**Recommendation**:

1. 10% 5‐aminolevulinicacid photodynamic therapy (5‐ALA‐PDT) is recommended for the treatment of flat warts. (1B)
2. Lasers and photodynamic therapy could be used to treat multiple flat warts. Lasers include CO_2_ laser, PDL (pulsed dye laser), and YAG laser. Photodynamic therapy (PDT) includes 5‐aminolevulinic acid (ALA) or 5‐methylaminolevulinic acid (MAL) photodynamic therapy. (2C)
3. Injection of bleomycin or *Candida albicans* antigen is recommended to treat flat warts. (1B)

**Summary of the evidence**:

Flat warts mostly appear in facial areas of children or young adults. It is commonly caused HPV type 3, 10, 28, and 41. There is no specific antiviral treatment thus far.^69^

A 2017 systematic review^70^ showed that compared with CO_2_ laser, ALA‐PDT had a lower recurrence rate, fewer adverse reactions, and a better prognosis for treating flat warts (*p* < 0.05). Compared with topical imiquimod cream, ALA‐PDT had a higher curative effect, also with a higher incidence of adverse reactions (*p* < 0.05). Compared with liquid nitrogen freezing, ALA‐PDT had a better curative effect with a lower recurrence rate and adverse reaction rate (*p* < 0.05). An RCT^71^ showed that 10% ALA was more effective than 5% and 20% ALA regarding the complete remission rate after 12 weeks (33.30% vs. 14.30% vs. 26.30%, *p <* 0.05). The rate of hyperpigmentation after 12 weeks was in the descending order of 33.30%, 15.60%, and 12.90% by use of 20%, 5%, and 10% ALA, respectively (*p* < 0.05).

A systematic review in 2016^72^ showed that CO_2_, PDL, and Nd: YAG are currently the most studied lasers for the treatment of nongenital verrucae with a response rate of 50.00−100.00% for CO_2_ laser, 47.10−100.00% for PDL, and 46.34−100.00% for Nd: YAG laser. PDL was comparable in effectiveness to traditional therapies such as cryotherapy and topical cantharidin. Combination of PDL with drugs such as bleomycin and salicylic acid had higher success rates, PDL had fewer adverse reactions compared with Nd: YAG or CO_2_ laser in the treatment of nongenital warts. In 2016, a case series study^73^ showed that long‐pulse 532 nm LP Nd: YAG laser achieved 92.00% complete removal of all warts after one course of treatment. Lesions with longer duration (over 2 years vs. fewer than 6 months) had lower clearance rates (84.00% vs. 98.00%, respectively).

The working panel of the guideline conducted a meta‐analysis of four RCTs^74^, ^75^, ^76^, ^77^ showing that the effective rate of intralesional injection of *Candida albicans* antigen to treat flat warts was significantly higher than those of other options such as oral isotretinoin, local injection of 5‐FU and other microbial antigens (*I* ^2^ = 77%, RR = 0.80, 95% CI [0.24, 2.72], *p <* 0.0001). While in one of the RCTs^77^, it showed that the cure rate of bleomycin injection was significantly higher than that of *Candida albicans* antigen as well as 5‐FU. Side effects included injection pain, local erythema and edema for all, and a few cases of flu‐like symptoms in *Candida albicans* antigen recipients.

**Question 9: What are the recommended clinical treatment methods for plantar warts, including multiple, recurrent, and refractory conditions?**

**Recommendation**:

1. Local hyperthermia is suggested for patients with plantar warts. (2B)
2. Cryotherapy is suggested for patients with plantar warts. (2B)
3. Long‐pulsed 1064 nm Nd: YAG laser combined with topical moisturizing cream treatment or optimized CO_2_ laser treatment is recommended for plantar warts. Local injections of recombinant human IL‐2 in combination with CO_2_ laser are recommended for recalcitrant plantar warts. (1B)
4. Local injections of bleomycin are recommended for the treatment of plantar warts. (1B)

**Summary of the evidence**:

The plantar wart is a common viral skin disease infected mostly by HPV types 1, 2, 4, 27, and 57, which is challenging because of the frequent recurrences after treatment.^78^

An RCT^79^ applied local hyperthermia at 44°C for 30 min in a session, in a protocol of application on day 1, 2, 3, 17, and 18, on a single target lesion. The results showed that 53.57% of patients with plantar warts and 11.54% of patients in the sham treatment group achieved complete cure (*χ* ^2^ = 10.718, *p* = 0.001), 3 months after completion of the treatment protocol. No recurrent case was reported in the study. Additional benefits were the reduction to 80.00% of loading‐bearing pain after the treatment and the removal of lesions at distant sites in successful cases. A cohort study^68^ treated plantar warts at local temperatures best tolerated by the patients which ranged from 43.5°C to 47.5°C (average at 45.3°C). By the end of 3 months after the therapy, 15 of 23 (65.22%) cases were cured. The treatment response was not correlated with wart number, sex, age, or temperatures applied.

An RCT^80^ showed that cryotherapy had a significantly higher complete cure rate than the silver tape closure treatment (58.00% vs. 20.00% out of 50 patients in each group) in the scheduled 8 weeks of study. There were no significant differences in response in either group in association with the duration of the disease. The selection of different cryotherapy devices may affect efficacy. An RCT^81^ concluded that nitrous oxide freezing had a higher cure rate in the treatment of common warts and plantar warts than the other two commercially available devices (dimethyl ether and propane freezing device and dimethyl ether without or with metal nib device, 82.00%, 47.37%, and 52.78%, respectively, *p* = 0.001). The patients treated were those with a disease duration of no more than 6 months.

A cohort study on 240 patients^82^ showed that the use of moisturizing cream before long‐pulse 1064 nanometer Nd: YAG laser treatment achieved a clearance rate of 97.08%, by an average treatment session of 1.3 (range 1–3), 75.80% cleared by one session of treatment.

An RCT^83^ on the treatment of recalcitrant warts showed that local injection of recombinant human IL‐2 combined with CO_2_ laser treatment had a higher effective rate 2 months after treatment and a lower recurrence rate than CO_2_ laser treatment, cryotherapy, topical fluorouracil ointment and local injection of recombinant human IL‐2 (94.00%, 78.00%, 56.00%, 32.00%, and 44.00%, respectively). An RCT^84^ showed that the effective rate of optimized CO_2_ laser (outputting grid spot) treatment and traditional CO_2_ laser treatment for plantar warts were 95.71% and 81.54%, respectively (*χ* ^2^ = 6.858, *p* < 0.05), after 6 months of follow‐up.

An RCT^85^ showed that the cure rate of intralesional bleomycin treatment were 63.02%, superior to that of 48.82% by cryotherapy (*p* < 0.05).

**Question 10: What are the recommended clinical treatment methods for CA, including multiple, recurrent, and refractory conditions?**

**Recommendation**:

1. Combination of 5% imiquimod with traditional physical therapy (lasers, cryotherapy) is recommended to treat CA. (1A)
2. Topical treatment is recommended for genital warts with single lesion size less than 5 mm or confluent lesion size less than 10 mm, or a total number of warts less than 15. (1B)
3. ALA‐PDT alone is recommended to treat genital warts <5 mm in size. (1B)
4. ALA‐PDT combined with traditional physical therapy is recommended to reduce the rate of recurrence. (1B)
5. Surgery is recommended for CA with pedicle or large volumes or recalcitrant. (1A)
6. Destructive physical therapy followed by immunomodulators (imiquimod or recombinant human interferonα−2b) or photodynamic therapy is recommended. (1B)
7. Topical 5% imiquimodor photodynamic therapy is considered for CA with underlining HIV infection. (2C)

**Summary of the evidence**:

The current principle of treatment of CA is to remove warts as early as possible, improve symptoms, eliminate subclinical infections and latent infections around warts, and reduce recurrence.^11^ The treatment methods of CA are divided into self‐application and office‐based treatment. Self‐application is mainly topical drugs, including podophyllotoxin and imiquimod. Office‐based treatment includes traditional physical therapy (CO_2_ laser, microwave, high‐frequency electric therapy, and liquid nitrogen freezing), photodynamic therapy, and trichloroacetic acid. Appropriate treatment methods should be based on the overall consideration of the condition of warts, patient selection, treatment cost and feasibility, side effects, and doctor's experience.

Traditional physical therapy has a quick clinical effect on hyperplastic lesions, but the effect of maintaining complete clearance is poor and the risk of recurrence is high.^86^ However, topical treatment with 5% imiquimod is helpful to achieve continuous clearance and reduce recurrence. The combination of these two can effectively clear CA and reduce CA recurrence.^87^ A systematic review in 2020^87^ showed that CO_2_ laser combined with 5% imiquimod had a higher clearance rate than CO_2_ laser alone (12 weeks after treatment: RR = 1.53, 95% CI [1.38, 1.71], *I* ^2^ = 46%; 24 weeks after treatment: RR = 1.90, 95% CI [1.42, 2.53], *I* ^2^ = 73%). The clearance rate of wart by electrocautery combined with 5% imiquimod was higher than that by electrocautery alone (RR = 1.62, 95% CI [1.33, 1.97], *I* ^2^ = 0%). Microwave combined with 5% imiquimod was better than microwave alone (RR = 2.20, 95% CI [1.26, 3.83], *I* ^2^ = 73%). 5% podophyllotoxin and 5% or 3.75% imiquimod cream are the most commonly used topical drugs. 5% podophyllotoxin tincture was topically applied twice a day for 3 consecutive days, followed by withdrawal for 4 days, 7 days as a course of treatment, with a maximum of 4 courses of treatment. In a network systematic review in 2020,^88^ six treatment options, including podophyllotoxin, imiquimod, tea polyphenol ointment, 5‐FU, cidofovir, and interferon cream were evaluated, setting the outcome index as complete removal of warts. 0.5% podophyllotoxin ointment or solution was the most effective in clearing the wart and the least recurrent. Meanwhile, in a network meta‐analysis^89^ in 2020, 0.5% podophyllotoxin (OR = 1.94, 95% CI [1.02, 3.71]) was significantly more efficacious than 5% imiquimod for lesion clearance

A 2019 systematic review^90^ showed that topical 0.5% podophyllotoxin solution was superior to 5% imiquimod cream in the treatment of patients with genital warts (OR = 0.07, 95% CI [0.001, 0.36]). In 2019, a systematic review^91^ showed that compared with the traditional tincture form of podophyllotoxin, podophyllotoxin nanogel could increase the cure rate (OR = 1.76, 95% CI [1.65,1.87], *p* < 0.00001), reduce the recurrence rate (OR = −0.32, 95% CI [−0.36,0.28], *p* < 0.00001), shorten the course of disease (95% CI [−9.41,9.14], *p* < 0.00001), reduce the adverse reactions such as edema (OR = 0.26, 95% CI [0.29, 0.22], *p* < 0.00001), erosion (OR = 0.25, 95% CI [0.34, 0.17], *p* < 0.00001), pain (OR = 0.35, 95% CI [−0.42,0.28], *p* < 0.00001), and effectively control HPV subclinical infection (OR = 0.46, 95% CI [0.31, 0.67], *p* < 0.00001).

Meta‐analysis of two RCTs^92^, ^93^ showed that the effective rate of topical imiquimod in the treatment of CA was significantly higher than those of other intervention groups (*I* ^2^ = 74.00%, RR = 1.60, 95% CI [1.35, 1.89], *p* = 0.009). In 2015, an RCT^94^ studied the clinical effects of 2.5% or 3.75% imiquimod cream in the treatment of male CA for 12 consecutive weeks. The results showed that the clearance rate (CR) of 3.75% imiquimod cream was higher than that of 2.5% imiquimod cream (CR: 18.60% vs. 14.30%, *p* < 0.05)

Photodynamic therapy, as with ALA‐PDT, was applied once a week. ALA‐PDT therapy had certain advantages in reducing the recurrence rate due to its direct inhibition effect on virus replication.^95^ A systematic review in 2013^96^ showed that the recurrence rate of ALA‐PDT was lower than CO_2_ laser and cryotherapy with liquid nitrogen for urethral anogenital warts (AGW) (RR = 0.25, 95% CI [0.18, 0.36], *p* < 0.05). The recurrence rate of cervical condyloma in ALA‐PDT was lower than that of CO_2_ laser (RR = 0.28, 95% CI [0.12, 0.65], *p* < 0.05). A multicenter RCT study^97^ showed that after being followed up for 12 weeks, the recurrence rate of AGW was significantly lower in the ALA‐PDT group (10.77% vs. 33.33%, respectively, *p* < 0.05) compared with CO_2_ laser group, and again in patients with urethral AGW the recurrence rate was lower in ALA‐PDT group than CO_2_ laser group (10.53% vs. 36.36%, respectively, *p* < 0.05). A multicenter retrospective study^98^ treated AGW patients using ALA‐PDT (once a week for 3 weeks) and assessed the efficacy after each treatment. The results showed increased clearance rates with more treatment sessions. Clearance rates after 1, 2, and 3 weeks of treatment were 68.12%, 81.16%, and 95.27%, respectively, *p* < 0.001). Small warts (<5 mm) had a higher clearance rate than warts of >5 mm (97.74% vs. 88.73%, *p* < 0.001). The recurrence rates varied at different locations of warts and perianal warts had the highest recurrence rates while those at labia had the lowest ones (30.23% vs. 11.54%, respectively). The adverse reactions included redness, pain, erosion, ulcer, and pigmentation, and the incidence rates were 7.72%, 8.10%, 2.26%, 0.94%, and 0.19%, respectively. There was no reported case of urethral malformation and sexual dysfunction. One clinical observation^99^ showed that after treatment for 1, 2, and 3 weeks, the clearance rate of warts with ALA‐PDT was higher than cryotherapy with liquid nitrogen at rates of 73.23% vs. 53.19% (at week 1), 85.83% vs. 63.83% (at week 2), and 93.70% vs. 70.21% (at week 3), all *p* < 0.01. After 3 months of follow‐up, the recurrence rate of ALA‐PDT was lower than cryotherapy with liquid nitrogen (10.17% vs. 45.45%, *p* < 0.01).

A systematic review in 2014^100^ revealed that ALA‐PDT combined with liquid nitrogen cryotherapy had a higher cure rate (OR = 4.82, 95% CI [3.33, 7.00], *p* < 0.01) and lower recurrence rate (OR = 0.32, 95% CI [0.21, 0.50], *p* < 0.01) compared with the liquid nitrogen freezing alone in patients with AGW. Two systematic review^96^, ^101^ showed that the local application of ALA‐PDT combined with CO_2_ laser had a lower recurrence rate than using CO_2_ laser alone after a 12 weeks follow‐up (RR = 0.20, 95% CI [0.09, 0.44], *p* < 0.05) in 13 RCTs and a 24 weeks follow‐up (RR = 0.23, 95% CI [0.11, 0.51], *p* < 0.05) in 7 RCTs. What's more, the combined treatment had a lower incidence and severity of adverse reactions, and patients were well tolerated.^101^ In a single‐arm clinical trial,^102^ 98 patients with warts were treated with laser ablation and then applied to three sessions of ALA‐PDT treatments. The results showed that 92 cases (93.88%) were completely cured. After 3 months of follow‐up, 18 (18.37%) cases had relapsed lesions near the treatment sites. A meta‐analysis of six RCTs^103^, ^104^, ^105^, ^106^, ^107^, ^108^ showed that the cure rate of ALA‐PDT combined with conventional physical treatments was higher than those of conventional physical treatment alone (*I* ^2^ = 89%, RR = 1.33, 95% CI [1.09, 1.63], *p* = 0.005). ALA‐PDT combined with conventional physical treatments had lower recurrence rates than those of conventional physical treatment alone, in 1 month (*I* ^2^ = 0%, RR = 0.20, 95% CI [0.13, 0.32], *p*<0.00001), 2 months (*I* ^2^ = 0%, RR = 0.16, 95% CI [0.11, 0.22], *p*<0.00001), and 3 months (*I* ^2^ = 0%, RR = 0.25, 95% CI [0.19, 0.34], *p*<0.00001).^101^, ^104^, ^105^

Surgical resection is suitable for the treatment of pedicled or massive warts, recalcitrant warts, and warts with repeated attacks in a short time. In a network meta‐analysis on anogenital warts in 2020,^88^ surgical resection achieved the best effect in removing the lesions (RR = 10.54, 95% CI [4.53, 24.52]), as compared with other methods like 5‐FU, ablation, ablation and imiquimod, cidofovir, 9% citric acid, or CO_2_ laser. A systematic review^90^ published in 2019, in which the outcome index was no recurrence of CA, showed that surgery had the best effect in reducing recurrence risk after thorough removal of CA compared with cryotherapy, 5% imiquimod cream, 0.5% podophyllotoxin solution, and 20–25% podophyllate.

Perianal CA is difficult to remove and the recurrence rate is high. The meta‐analysis results of two RCTs^109^, ^110^ showed the effective rate of the combined treatment group (recombinant human interferon α−2b injection combined with liquid nitrogen cryotherapy or CO_2_) was significantly higher than that of liquid nitrogen cryotherapy or CO_2_ group alone (*I* ^2^ = 85%, RR = 1.47, 95% CI [1.04, 2.06], *p* = 0.03) and the recurrence rate was lower (*I* ^2^ = 89%, RR = 1.33, 95% CI [1.09, 1.63], *p* = 0.005).

AGW concurrent with HIV infection is more prone to malignant transformation and more difficult to treat. Traditional therapy such as CO_2_ laser, microwave, high‐frequency electric therapy, and liquid nitrogen freezing treatment often fails and the recurrence rate is high. An RCT^111^ compared the efficacy of ALA‐PDT with topical 5% imiquimod cream for AGW with HIV infections, along with standard anti‐HIV therapy during the trial. ALA‐PDT had a higher cure rate (84.00% vs. 52.00%, *p* < 0.05), lower recurrence rate (16.00% vs. 48.00%, *p* < 0.05), and reduced incidence of adverse reactions related to skin and mucosal injury (*p* < 0.05), but the treatment‐related pain was more severe (*p* < 0.05). A case‐series study^112^ observed the cure rate 20 weeks after treatment with microwave therapy combined with imiquimod cream topical application (topical application after 1 week of microwave treatment) in AGW patients concurrent with HIV infection. The results showed that in patients with CD4^+^T lymphocyte number ≥350 × 10^6^/L, the cure rate was 89.55%, and CD4^+^ T lymphocyte below that number was 72.09%. A cohort study^113^ focusing on different methods for the treatment of patients with anal CA complicated with HIV infection showed that 1 year after treatment, the cumulative recurrence rates were 6.15% (4/65, 95% CI [2, 15], error data in original study) by electroresection, 11.11% (3/27, 95% CI [4, 28]) by infrared coagulation, and 11.11% (1/9, 95% CI [2, 44]) by imiquimod. After 10 years of follow‐up, cumulative recurrence rates were 46.15% (95% CI [35, 58]) by electroresection, 55.56% (95% CI [37, 72]) by infrared coagulation, 55.56% (95% CI [27, 81]) by imiquimod, and 50.00% (1 case, 95% CI [1, 91]) by cryotherapy.

**Question 11: What are the recommended clinical treatment methods for epidermodysplasia verruciformis (EV)?**

**Recommendation**:

1. 1.5‐FU and imiquimod could be used topically for early stage EV with lesions few in numbers and small in size. (2D)
2. Tretinoic acids could be taken orally and topically when the lesions spread all over the body. (2D)
3. Refractory lesions could be treated with electrocautery and cryotherapy. (2D)
4. Surgery removal is suggested for patients with severe keratinized lesions, precancerous lesions, and squamous cell carcinoma. (2D)
5. All patients need sun protection education and guidance on sun protection. (1D)

**Summary of the evidence**:

Verrucous epidermal dysplasia is a rare chronic disease characterized by genetic susceptibility to HPV, manifesting as highly pleomorphic and disseminated lesions. Solar keratosis frequently occurs after 30 years of age, and half of them evolve into squamous cell carcinoma. There is currently no specific treatment, and different treatment methods are chosen according to the manifestations of the skin lesions. Above all, sun protection is necessary to prevent malignant transformation.

In a case report, two 23‐year‐old female patients with multiple flat EV lesions on the face were topically applied 5‐FU combined with imiquimod once a day and three days per week, and the area and numbers of warts were reduced, while recurrent after 1 year.^114^ A 40‐year‐old female EV patient with generalized lesion was treated with oral Isotretinoin combined with Tazarotene gel, and the lesions subsided significantly (no long‐term follow‐up).^115^ A 16‐year‐old female patient with generalized skin lesions on the face and neck was treated with systemic ganciclovir, BCG polysaccharide nucleic acid, and the skin lesions were treated with electrocautery and cryotherapy. A proportion of the lesions were removed while there were remaining ones on several sites of the body.^116^ A 50‐year‐old male patient with severe hyperkeratotic plaques on the face and limbs was diagnosed with clinical manifestation and HPV 51 DNA positivity. Surgical resection removed the plaques over his hands. There was no recurrence in a 1‐year follow‐up.^117^ A case report described a 56‐year‐old female patient with brown flat verrucous papules all over the body for 40 years. She developed multiple lesions histologically confirmed Bowen's disease, which was surgically removed.^118^

**Question 12: How should children (under the age of 18) with cutaneous warts be treated?**

**Recommendation**:

1. Intralesional *Candida* antigen immunotherapy is suggested for children with recalcitrant and multiple warts. (2C)
2. Children with CA could be treated with 5‐ALA ‐PDT combined with high‐frequency electrocautery. (2C)
3. Children with recalcitrant CA around the anus could be treated with local thermotherapy. (2D)
4. Children with common warts can be treated with 5% imiquimod cream. (1B)
5. Monochloroacetic acid (MCA) is recommended for children with plantar warts. (1B)

**Summary of the evidence**:

Cutaneous warts are estimated to occur in up to 10% of children and young adults, with the greatest incidence between 12 and 16 years of age. Warts occur more frequently in girls than in boys. Common warts represent 70% of skin warts and occur primarily in children, whereas plantar and flat warts occur among slightly older populations.^119^

The first‐line treatment of skin warts includes topical use of salicylic acid and cryotherapy, but cryotherapy may cause pain and blisters and require repeated treatment, which limits its use in children. Intralesional immunotherapy is a promising method for the treatment of multiple or refractory warts in children, which can remove distant warts with minimal side effects, with cure rates of 23.30–95.20%.^120^

An RCT^121^ compared the efficacy of MMR (measles, mumps, and rubella) vaccine (*n* = 15), *Candida* antigen intralesion injection (*n* = 15), and saline control (*n* = 10) in 40 cases of children with anogenital warts. Of 15 patients in the treatment groups, 73.33% (MMR) and 80.00% (*Candida* antigen) achieved complete clearance, respectively, compared with saline control (1 out of 10 patients, 10.00%). There was no statistically significant difference between the MMR vaccine and the *Candida* antigen group. Adverse reactions were mild, and there was no recurrence after 6 months of follow‐up.

An RCT^122^ compared the efficacy of high‐frequency electrocautery combined with 5‐ALA‐PDT and high‐frequency electrocautery alone for treating CA in children. The result showed that the effective rates were not statistically different between the two groups (90.91% vs. 88.89%, *p* > 0.05), while the combination method had a significantly lower recurrence rate than the high‐frequency electrocautery group alone (13.64% vs. 44.44%, *p* < 0.05).

A case report showed^123^ that local thermotherapy at 44°C successfully cured a 2.5‐year‐old child with multiple CA around the anus, who failed several previous treatments. The treatment started once a day for 3 consecutive days, each treatment for 30 min, plus two sessions of treatment 2 weeks later. Then the treatment was conducted once a week for 5 weeks when the volume of the body of the wart started to decrease. Two more additional treatments were given, and in 4 months, all the lesions disappeared without recurrence the months to follow.

An RCT^124^ compared the effect of MCA and cryotherapy on common warts. The cure rates were 40/92(43.48%, 95% CI [34, 54]) for MCA and 50/93 (53.76%, 95% CI [44, 64]) for cryotherapy (risk difference (RD): 10%, 95% CI [−25, 4.0], *p* = 0.16), at week 13th after initiation of treatment. MCA could effectively replace cryotherapy, which could avoid pain and reduce blisters during treatment but could not avoid pain after treatment. In regard to plantar wart, cure rates were 49/106 (46.23%, 95% CI [37, 56]) for MCA and 45/115(39.13%, 95% CI [31, 48]) for cryotherapy combined with self‐daily applied salicylic acid (RD 7.10%, 95%CI [−5.9, 20], *p* = 0.29).MCA had similar efficacy but it could reduce the pain, blister, and burden of treatment.

**Question 13: How should pregnant women with cutaneous warts be treated?**

**Recommendation**:

1. Podophyllotoxin and imiquimod are not recommended for CA during pregnancy, but trichloroacetic acid can be used. (1A)
2. CA during pregnancy could be treated by liquid nitrogen cryotherapy or surgery. (2C)
3. Cesarean section is recommended when large warts may block the birth canal or cause massive bleeding. (1C)
4. CA during pregnancy could be treated with local thermotherapy. (2C)

**Summary of the evidence**:

Compared with the nonpregnancy patient, CA during pregnancy has rapid growth, a high recurrence rate and is easy to ulcerate and bleed, possibly due to changes in hormone levels in pregnant women, a genital environment conducive to HPV reproduction, limited clinical medication, and decreased immune function.^125^

Chinese guidelines for the diagnosis and treatment of CA in 2014^10^ and an expert consensus in 2017^11^ recommended that pregnant women with CA should be treated as early as possible. Podophyllotoxin and imiquimod were not recommended.^13^ Podophyllotoxin and imiquimod were prohibited during pregnancy because their fetal teratogenic effect was grade C for pregnancy medication classified by FDA.^126^ However, a cohort study^127^ showed that there was no statistical relationship between podophyllotoxin exposure and nonexposure groups in birth defects, spontaneous abortion, preterm delivery, and stillbirth. The study concluded that the use of podophyllotoxin during pregnancy (the dose of podophyllotoxin was not specified in this cohort study, and exposure was based on the prescription of the drug) did not increase the risk of adverse fetal outcomes. However, sufficient evidence from RCT trials on podophyllotoxin use in pregnant women are needed. Similarly, although medical reports^128^, ^129^, ^130^, ^131^ indicated that the use of imiquimod during pregnancy did not show adverse fetal outcomes, there was still a lack of large‐scale human trials, so it was not recommended as a first‐line drug for pregnant women.^132^ A comparative study^133^ showed that by combination therapy of CO_2_ laser and 85% trichloroacetic acid in treating 32 pregnant women with genital condylomas, 96.88% patients (31/32) achieved successful eradication with only maternal postoperative complications being pyelonephritis (1/32, 3.13%). The differences in obstetric complications such as premature rupture of membranes, premature onset of labor, and cesarean delivery between the case group (*n* = 32) and control group (*n* = 64) were not significant.

A retrospective study^134^ showed that liquid nitrogen cryotherapy combined with proanthocyanidins dressings (2–3 times a day for 1 week, 20 min for each dressing) cleared all visible CA in 46 pregnant women, with the recurrence rates of 2.17% in 1 month and 10.87% in 3 months.

The existence of warts rarely changed the mode of production, and cesarean section could be performed only when warts block the birth canal. The only serious complication of transvaginal delivery was respiratory papillomatosis in infants and young children, which was relatively rare (4/million).^135^

A report^136^ applied hyperthermia at 44°C for 30 min a day, 3 days in a row, and 2 sessions of treatment after a week. CA in two cases of pregnant women had been cleared 4 and 5 weeks after the last treatment, respectively. No apparent side effects were reported except slight burning pain. There was no recurrence during the 6‐month follow‐up.

## 4 DISCUSSION

### 4.1 Summary

This guideline covers several main types of cutaneous and mucosal HPV infectious diseases including common warts, flat warts, plantar warts, genital warts, and EV. Treatment for the special population such as children and pregnant women was also involved. Some clinical questions have been redefined and explained. The guideline aims to systematically and effectively guide the clinical management of warts, achieve better clinical outcomes for patients with HPV infectious skin diseases, improve the overall levels of medical services for skin warts, and reduce medical costs and economic burden.

### 4.2 Dissemination, implementation, and evaluation

Once the guideline is released, the guideline development team will mainly disseminate and promote the guideline in the following ways: (I) introduce it in relevant academic conferences; (II) organize guideline promotion special sessions in a planned way to ensure that medical practitioners fully understand and correctly apply the guidelines; (III) conduct research in the next 2 years to understand the dissemination of the guidelines and evaluate the impact of the implementation of the guidelines on clinical decision‐making and patient outcomes. However, as a rule, the choice of a treatment method is decided on consensus between the practitioner and the patient, as well as practitioners’ evaluation of the specificity of a patient. The strength of the recommendation and levels of evidence may offer certain help.

### 4.3 Strengths and limitations

The guidelines for HPV infectious skin diseases are formulated by evidence‐based methods. The guideline working groups have strictly followed the standards and procedures of evidence‐based guidelines formulation required by international organizations, selected specific problems in the clinical practice of HPV infectious diseases, and conducted a systematic search. The evidence body is formed after a systematic search and assessment, and based on patient preferences and values, as well as cost‐benefit factors, combined with the practical experience of multidisciplinary clinical experts. A high‐quality evidence‐based guideline has been formulated with evidence‐based medicine support, patient‐based, clinical problem‐oriented features.

Meanwhile, limitations exist such as low‐level evidence support of certain recommendations. Especially for EV, the included literature is mainly case reports so recommendations are formulated restrictively with less representativeness. For responders of the survey, specialists at the Department of Obstetrics and Gynecology were not concluded while they were also clinical practitioners of this guideline. Due to the cross‐disciplines nature, treatments of HPV infectious skin diseases have not included HPV vaccines. Only literature in English and Chinese were covered in the guideline and exclusion of literature in other languages may cause bias.

## 5 CONCLUSIONS

This guideline covers aspects of the diagnosis and treatment of cutaneous warts such as diagnostic gold standard, transmission routes, laboratory tests, treatment principle, clinical cure criterion, definitions, and treatments of common warts, flat warts, plantar warts, CA, and EV. Recommendations about special populations such as children and pregnant women are listed. It is a comprehensive and systematic evidence‐based guideline and we hope this guideline could systematically and effectively guide the clinical practice of cutaneous warts and improve the overall levels of medical services. We will update this guideline around 2025 according to the requirement of the international guide.

## CONFLICT OF INTEREST

All members of this guideline working group have signed a conflict of interest declaration form when determining to participate in the guideline work. All authors have confirmed that they have no potential conflicts of interest.

## WORKING TEAM

Steering committee: Xinghua Gao (The First Hospital of China Medical University), Yaolong Chen (Evidence‐Based Medicine Center, School of Basic Medical Sciences, Lanzhou University), Hongduo Chen (The First Hospital of China Medical University), Wanqing Liao (Shanghai Changzheng Hospital), Qianjin Lu (The Second Xiangya Hospital of Central South University), Jie Zheng (Ruijin Hospital), Jianzhong Zhang (Peking University People's Hospital), Xuejun Zhang (Anhui Medical University), and Jinghe Lang (Peking Union Medical College Hospital).

Consensus expert group: Hao Cheng (Sir Run Run Shaw Hospital), Lingyu Fu (The First Hospital of China Medical University), Xinghua Gao (The First Hospital of China Medical University), Heng Gu (Chinese Academy of Medical Sciences and Peking Union Medical College), Li He (First Affiliated Hospital of Kunming Medical University), Hongzhong Jin (Peking Union Medical College Hospital), Quanzhong Liu (Tianjin Medical University General Hospital), Lin Ma (Beijing Children's Hospital), Ruiqun Qi (The First Hospital of China Medical University), Youlin Qiao (Peking Union Medical College), Yuping Ran (West China Hospital), Zhu Shen (Sichuan Academy of Medical Sciences and Sichuan Provincial People's Hospital), Ping Song (Guang'anmen Hospital), Gang Wang (Xijing Hospital), Xiuli Wang (Shanghai Skin Disease Hospital), Yan Wu (The First Hospital of China Medical University), Shengxiang Xiao (The Second Affiliated Hospital, School of Medicine, Xi'an Jiaotong University), Jinhua Xu (Huashan Hospital), Huilan Yang (General Hospital of Southern Theatre Command of PLA), Rongya Yang (General Hospital of Beijing Military Command of PLA), Furen Zhang (Shandong Provincial Hospital for Skin Diseases & Shandong Provincial Institute of Dermatology and Venereology), Kang Zeng (Nanfang Hospital), Ping Tu (Peking University First Hospital), Yuzhen Li (Second Affiliated Hospital of Harbin Medical University), Xiang Chen (Xiangya Hospital), Aijun Chen (The First Affiliated Hospital of Chongqing Medical University), Junling Zhang (Tianjin Academy of Traditional Chinese Medicine Affiliated Hospital), Shanshan Li (The First Hospital of Jilin University), Zhirong Yao (Xinhua Hospital), Weihua Pan (Second Affiliated Hospital of Naval Medical University), Songmei Geng (The Second Affiliated Hospital of Xi'an Jiaotong University), and Juan Tao (Union Hospital, Tongji Medical College, Huazhong University of Science and Technology).

Secretarial group: Peiyao Zhu, Yang Yang, Xu Han, Yiping Zhao, Dongxin Shi, Yunqiu Gao (The First Hospital of China Medical University), Jianjian Wang, Qiangqiang Guo, Shouyuan Wu, Hui Lan, and Juanjuan Zhang (Evidence‐Based Medicine Center, School of Basic Medical Sciences, Lanzhou University).

Evidence evaluation group: Xueli Niu, Yao Lu, Tianxin Cong, Yanjun Li, Congcong He, Xinyun Fan, Ze Wu, Wenzhen Hu, Kangle Fu, Yining Wang, Qu Qi, Donghong Sun, Xiaoxue Yang, Jiali Yin, Yifei Liu, Yiping Zhao, Gaiyang Xu, Mingsui Tang, Chang Fu, Jingyi Li, Weibao Lu, Qixin Han, Shuzhen Ren, Dongxin Shi, Sitong Liu, Meihui Shi, Weitong Yan, Peihong Sun, Jing Zeng, Chengfei Zhuang, Jingyu Wang, Peiyao Zhu, and Xu Han (The First Hospital of China Medical University).

External review group: Xing‐Hua Gao, Rui‐Qun Qi, Yang Yang and Peiyao Zhu (The First Hospital of China Medical University), Yaolong Chen, Jianjian Wang, Shouyuan Wu, Qiangqiang Guo, Hui Lan, and Juanjuan Zhang (Evidence‐Based Medicine Center, School of Basic Medical Sciences, Lanzhou University).
